# Risk of Systemic Health Events and Mortality After Vitrectomy for Diabetic Retinopathy in Patients with Type 2 Diabetes

**DOI:** 10.1016/j.xops.2025.100880

**Published:** 2025-07-07

**Authors:** Dane A. Jester, Muhammad Z. Chauhan, Zain S. Hussain, Sam Karimaghaei, Jawad Muayad, Asad Loya, Ahmed F. Shakarchi, Ahmed B. Sallam

**Affiliations:** 1 Harvey and Bernice Jones Eye Institute, University of Arkansas for Medical Sciences, Little Rock, Arkansas; 2 School of Medicine, Texas A&M University, Houston, Texas; 3 Cullen Eye Institute, Baylor College of Medicine, Houston, Texas

**Keywords:** Diabetes mellitus, Diabetic retinopathy, Pars plana vitrectomy, Retrospective cohort study, TriNetX research network

## Abstract

**Purpose:**

To quantify the risk of mortality, myocardial infarction (MI), stroke, and amputation in patients with type 2 diabetes mellitus (T2DM) who underwent pars plana vitrectomy (PPV) for diabetic retinopathy (DR) compared with those not requiring PPV.

**Design:**

A retrospective cohort study utilizing the TriNetX US Collaborative Network.

**Subjects:**

The study included 9081 patients with T2DM who underwent PPV for DR, 363 116 patients with T2DM with DR but no PPV, 92 645 patients with T2DM without DR, and 3 264 709 healthy individuals, all aged ≥18 years.

**Methods:**

We identified cohorts using specific International Classification of Diseases, 10th Revision and Current Procedural Technology codes. We used propensity score matching to adjust for covariates including age, gender, race, ethnicity, systemic pathology, and ocular conditions unrelated to diabetes.

**Main Outcome Measures:**

The primary outcome measures were the hazard ratios (HRs) for mortality, MI, stroke, and amputation at 1, 3, and 5 years after PPV compared with the control groups.

**Results:**

Patients with T2DM undergoing PPV for DR had higher risk of systemic events and mortality. Compared with patients with DR not requiring PPV, the PPV cohort had a higher risk at 1 year for stroke (HR: 1.51; 95% confidence interval [CI]: 1.03, 2.21) and amputation (HR: 1.85; 95% CI: 1.08, 3.16). At 3 years, the risks for MI (HR: 1.44; 95% CI: 1.17, 1.78), stroke (HR: 1.61; 95% CI: 1.25, 2.07), and amputation (HR: 2.17; 95% CI: 1.54, 3.05) were significantly elevated. At 5 years, the risks for mortality (HR: 1.28; 95% CI: 1.13, 1.43), MI (HR: 1.50; 95% CI: 1.26, 1.78), stroke (HR: 1.54; 95% CI: 1.25, 1.91), and amputation (HR: 2.10; 95% CI: 1.58, 2.81) were all significantly higher. When compared with diabetic patients without DR or healthy patients, the PPV cohort faced higher risk of each health outcome analyzed at intervals of 1, 3, and 5 years.

**Conclusions:**

We found a significant association between patients with T2DM with DR requiring PPV and an increased risk of mortality, MI, stroke, and amputation compared with non-PPV patients with DR, diabetics without DR, and healthy individuals. These findings underscore the need for monitoring and management of systemic health in diabetic patients undergoing PPV for advanced DR.

**Financial Disclosure(s):**

The author(s) have no proprietary or commercial interest in any materials discussed in this article.

Diabetes mellitus (DM) is a metabolic disease that is prevalent and damaging across the global population. Type 2 DM (T2DM), the most common subtype, is characterized by hyperglycemia due to insulin resistance and eventually diminished insulin production.^1^ Among the predominant complications of diabetes is diabetic retinopathy (DR), which is the most common retinal vascular disease,^2^ affecting >100 million people worldwide.^3^ This sight-threatening pathology stems from hyperglycemic and ischemic damage to the retinal microvasculature and is classified as either nonproliferative DR or proliferative DR, with the latter defined by the presence of retinal neovascularization. In most patients, DR can be adequately managed with systemic control of glucose levels, intravitreal pharmacological therapy, or panretinal photocoagulation laser.^4^ However, in cases of advanced proliferative DR complicated by nonclearing vitreous hemorrhage or tractional retinal detachment, pars plana vitrectomy (PPV) surgery may be indicated to restore vision. The development of these complications reflects severe underlying hyperglycemia and ischemia affecting the retinal vasculature.^5^

Beyond the retinal complications, longstanding hyperglycemia is associated with a number of systemic health events, including mortality,^6^^,^^7^ myocardial infarction (MI),^8^^,^^9^ stroke,^8^^,^^10^ and amputation.^11^^,^^12^ Given this association, the presence and progression of DR, especially in cases requiring PPV, are likely reflective of an increased risk of these events. Prior publications have linked DR to increased incidence of mortality,^13^, ^14^, ^15^ MI,^14^^,^^16^, ^17^, ^18^, ^19^, ^20^ stroke,^21^, ^22^, ^23^ and amputation,^24^, ^25^, ^26^ among other complications. Regarding the subset of patients requiring PPV, a number of publications describe single-cohort analyses examining mortality rates in this population.^27^, ^28^, ^29^, ^30^, ^31^, ^32^, ^33^, ^34^, ^35^ While these studies are in consensus regarding an increased incidence of mortality after diabetic PPV, the extent of this increase varied significantly. Additionally, previous studies were limited by their methodology, reporting only survival rates without comparison to a control cohort and utilizing small sample sizes. In this study, we aimed to use a large US national cohort to quantify the risk of mortality, MI, stroke, and amputation in patients requiring PPV compared with those who did not undergo diabetic PPV.

## Methods

### Data Collection

We conducted this exploratory, retrospective study using the TriNetX database, a large, global research network. We collated the study data in October 2024, from the TriNetX US Collaborative Network, which provided access to deidentified electronic medical records (diagnoses, procedures, medications, laboratory values, genomic information) from approximately 118 million patients from 68 health care organizations in the United States. We employed a study period of January 1, 2005 to January 1, 2024. This retrospective study was exempted from informed consent. The data we utilized for secondary analysis were previously existing, did not involve intervention or interaction with human subjects, and were deidentified. Our methods adhered to the tenets of the Declaration of Helsinki. Given that we utilized no identifiable patient information, the University of Arkansas Institutional Review Board determined that approval was not required.

### Cohort Design

We assembled 4 cohorts: patients who have undergone PPV for DR, patients with DR not requiring PPV, patients with DM but no DR, and healthy nondiabetic patients. To assemble these cohorts, we used International Classification of Diseases, 10th Revision (ICD-10), Current Procedural Technology (CPT), and Systematized Nomenclature of Medicine codes. Current Procedural Technology codes, which are vital to our study design, are primarily used in the United States, so it was imperative that we utilized the US Collaborative Network as opposed to international TriNetX networks.^36^^,^^37^

### Inclusion and Exclusion Criteria

Because each subtype of DM is driven by distinct pathophysiology and accompanied by differing health risks, we opted to include only patients affected by a single subtype—T2DM.^38^ To ensure only adult patients were featured in our analysis, we excluded patients <18 years old. We required patients in the diabetic PPV cohort to have a record of CPT codes 67040, 67041, 67042, or 67113 for PPV surgery within the study period. To isolate a cohort of patients with T2DM with PPV for complications of DR as opposed to other etiologies, we required an ICD-10 code for retinopathy related to T2DM (E11.31-35) as well as a diagnosis of tractional retinal detachment (H33.4) or vitreous hemorrhage (H43.1) on the day of surgery. We excluded patients with a retinal detachment stemming from a retinal break (H33.0) in the 3 months prior to PPV to ensure only patients with PPV for DR were in the cohort. Coding utilized for cohort design is shown in Table S1 (available at www.ophthalmologyscience.org).

For the DR with no PPV cohort, we included patients with a record of retinopathy related to T2DM and excluded those with a record of PPV. We assembled the DM with no DR cohort using the ICD-10 code for T2DM (E11), excluding those with the aforementioned DR or PPV codes. In addition, we required patients in this group to have a documented ophthalmic exam (ICD-10 codes Z01.0 or Z13.5 or Systematized Nomenclature of Medicine code 722161008) after diagnosis of diabetes to minimize the possibility of patients having undocumented DR. We compiled the “healthy” cohort using the ICD-10 code Z00.00—“encounter for general adult medical examination without abnormal findings.” We excluded patients with a record of T2DM, retinopathy related to T2DM, or PPV. Patients in the DM without DR and healthy cohorts are likely to undergo significantly less frequent follow-up in comparison to the 2 diabetic eye disease cohorts, providing an opportunity for skewing of data. To counter this discrepancy, we required patients in these cohorts to have documented clinical visits in the 1 to 5 years after and ≥5 years after the index date, aside from instances of mortality during this time. Finally, we excluded patients with a prior record of the health events we investigated (as defined later) from all cohorts to ensure the outcomes recorded were only first incidences. We opted to analyze only first instances because a previous history of these systemic events would be an impactful confounding variable, potentially obscuring the association we aimed to examine.

### Propensity Score Matching

To eliminate the effects of remaining confounding variables, we conducted propensity score matching (PSM) between the diabetic PPV cohort and the cohort of comparison for each analysis with TriNetX’s integrated program, utilizing 1:1 matching by nearest neighbor greedy matching algorithm. Propensity score matching covariates were unique in each comparison based on the level of diabetic disease present. Utilized covariates include age, gender, ethnicity, race, A1c, body mass index, nicotine dependence, alcohol abuse, systemic pathology (hypertension, chronic obstructive pulmonary disease, chronic kidney disease, hyperlipidemia), medication (insulin, antihypertensives, antilipemics), and ocular pathology (stages of DR, cataract, glaucoma, macular degeneration, lattice degeneration). Matching for ocular pathology not directly related to DM was an important facet of our design, given that patients with other pathology are more likely to regularly engage with eye care providers, increasing the likelihood of accurate diagnosis and appropriate care. This matching helped to create similar levels of care-engagement across cohorts.

### Study Outcomes

We assessed the risk of 4 systemic health events—mortality, MI, stroke, and amputation—after PSM. We defined mortality using ICD-10 code R99 (“ill-defined and unknown cause of mortality”) or TriNetX categorization (“deceased”). We included MIs with or without ST elevation (I21 or I22), and we defined stroke using code I63 (“cerebral infarction”). For amputation, we utilized a number of CPT codes involving the hands, feet, and legs, as shown in Table S2 (available at www.ophthalmologyscience.org).

### Statistical Analysis

After PSM, we estimated hazard ratios (HRs) at 1, 3, and 5 years after the index date to compare the risk of each outcome between PPV patients with diabetes and patients with DR not requiring PPV, patients with DM without DR, and patients without DM. We used the term “index event” to describe the point at which a patient fulfills all cohort inclusion criteria, while still avoiding all exclusion criteria. We elected to use HR because this measure incorporates time-to-event and is best equipped to measure censored data. We reported all HRs with accompanying 95% confidence intervals (CIs) and conducted all analyses using TriNetX’s platform-integrated analytics program.

## Results

### Cohorts Characteristics

Before PSM, a total of 9081 patients met the criteria to be included in the diabetic PPV cohort. Patients in this group had a mean age of 54.5 years at the time of PPV, and 45.2% were female. For the DR without PPV cohort, 363 116 patients met the inclusion criteria. These patients were an average age of 60.5 years at the time of diagnosis, and 48.7% were female. In the DM without DR cohort, which included 92 645 patients, the average age at the index date was 63.0 years, and 50.6% were female. Finally, 3 264 709 patients were in the healthy cohort. The average age at the time of the well visit was 50.8 years, and 58.7% were female. In all 4 cohorts, “White” was the most common race, followed by “Black or African American.”

### DR Requiring Vitrectomy vs. DR without Vitrectomy

**Figure 1:**
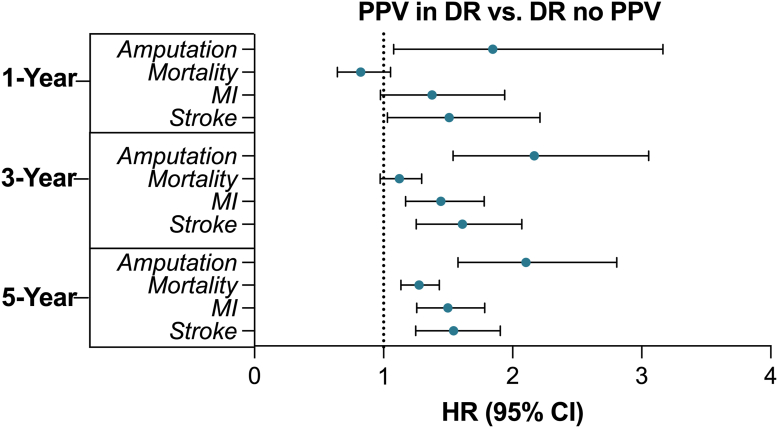
Forest plot of HR at 1, 3, and 5 years for mortality and systemic outcomes between patients with type 2 diabetes with DR undergoing PPV compared with those with DR without PPV. CI = confidence interval; DR = diabetic retinopathy; HR = hazard ratio; MI = myocardial infarction; PPV = pars plana vitrectomy.

Table 1 details the baseline characteristics and PSM inputs and results for the comparison between patients with T2DM who underwent PPV for DR and those with DR not requiring PPV. The mean (standard deviation) follow-up in days at the end of the 5-year window was 1075.1 (717.1) days in the PPV cohort and 1201.3 (700.9) days in the DR no PPV cohort. In the matched PPV cohort, the 5-year survival probability was 79.7%. One year after the index date, HRs in this comparison were significantly higher for stroke, at 1.51 (95% CI: 1.03, 2.21) and amputation, at 1.85 (95% CI: 1.08, 3.16). One-year HRs were not significantly different for mortality or MI. In the 3-year analysis, HRs were significantly elevated for MI, at 1.44 (95% CI: 1.17, 1.78), stroke, at 1.61 (95% CI: 1.25, 2.07), and amputation, at 2.17 (95% CI: 1.54, 3.05). The 3-year HR for mortality was not significantly different between cohorts. Five years after the index date, HRs were significantly higher in each systemic event examined, at 1.28 (95% CI: 1.13, 1.43) for mortality, 1.50 (95% CI: 1.26, 1.78) for MI, 1.54 (95% CI: 1.25, 1.91) for stroke, and 2.10 (95% CI: 1.58, 2.81) for amputation. Of note, for each event, the comparative risk was higher at 5 years than 1 year (Fig 1).

**Table 1:** Propensity Score Matching Results between Patients with Type 2 Diabetes with DR Requiring Vitrectomy and DR without Vitrectomy

Characteristics	Before Matching		Std. Diff.	After Matching		Std. Diff.
	DR Requiring Vitrectomy (n = 9081)	DR without Vitrectomy (n = 363 116)		DR Requiring Vitrectomy (n = 4822)	DR without Vitrectomy (n = 4822)	
Age, mean (SD)	54.5 (12.2)	60.5 (13.6)	0.467	56.3 (12.4)	57.5 (13.6)	0.092
Gender, No. (%)						
Male	4714 (51.91%)	172 142 (47.41%)	0.090	2407 (49.92%)	2400 (49.77%)	0.003
Female	4105 (45.20%)	176 997 (48.74%)	0.071	2175 (45.11%)	2176 (45.13%)	<0.001
Unknown	262 (2.89%)	13 977 (3.85%)	0.054	240 (4.98%)	246 (5.10%)	0.003
Ethnicity, No. (%)						
Non-Hispanic or Latino	4811 (52.98%)	216 499 (59.62%)	0.134	2623 (54.40%)	2570 (53.30%)	0.022
Hispanic or Latino	2421 (26.66%)	56 316 (15.51%)	0.276	890 (18.46%)	625 (12.96%)	0.152
Unknown ethnicity	1849 (20.36%)	90 301 (24.87%)	0.108	1309 (27.15%)	1627 (33.74%)	0.144
Race, No. (%)						
White	4502 (49.58%)	188 564 (51.93%)	0.047	2262 (46.91%)	2254 (46.74%)	0.003
Black or AA	1958 (21.56%)	76 677 (21.11%)	0.011	1047 (21.71%)	1064 (22.07%)	0.009
Asian	274 (3.02%)	15 216 (4.19%)	0.063	170 (3.53%)	155 (3.21%)	0.017
American Indian or Alaskan Native	87 (0.96%)	2625 (0.72%)	0.026	39 (0.81%)	25 (0.52%)	0.036
Native Hawaiian or Pacific Islander	152 (1.67%)	6948 (1.91%)	0.018	115 (2.39%)	88 (1.83%)	0.039
Other race	766 (8.44%)	19 435 (5.35%)	0.122	303 (6.28%)	232 (4.81%)	0.064
Unknown race	1342 (14.78%)	53 651 (14.78%)	<0.001	886 (18.37%)	1004 (20.82%)	0.062
Comorbidities, No. (%)						
Essential hypertension	5273 (55.07%)	198 523 (54.67%)	0.069	2496 (51.76%)	2220 (46.04%)	0.115
Hyperlipidemia	3020 (33.26%)	139 383 (38.39%)	0.107	1468 (30.44%)	1262 (26.17%)	0.095
Chronic kidney disease	2428 (26.73%)	68 445 (18.85%)	0.189	1094 (22.69%)	942 (19.54%)	0.077
COPD	271 (2.98%)	19 628 (5.41%)	0.121	156 (3.24%)	127 (2.63%)	0.036
Alcohol abuse	162 (1.78%)	6036 (1.66%)	0.009	71 (1.47%)	62 (1.29%)	0.016
Nicotine dependence	792 (8.72%)	29 421 (8.10%)	0.022	369 (7.65%)	312 (6.47%)	0.046
BMI <18.5	145 (1.60%)	4910 (1.35%)	0.020	57 (1.18%)	37 (0.77%)	0.042
BMI ≥18.5, <25	871 (9.59%)	29 152 (8.03%)	0.055	409 (8.48%)	359 (7.45%)	0.038
BMI ≥25, <30	1445 (15.91%)	54 526 (15.02%)	0.025	738 (15.31%)	677 (14.04%)	0.036
BMI ≥30	1967 (21.66%)	82 468 (22.71%)	0.025	1042 (21.61%)	951 (19.72%)	0.047
Laboratory value, No (%)						
HbA1C ≥6.5	3594 (39.58%)	152 822 (42.09%)	0.051	1818 (37.70%)	1639 (33.99%)	0.078
HbA1C <6.5	1155 (12.72%)	71 738 (19.76%)	0.192	630 (13.07%)	574 (11.90%)	0.035
Medication, No. (%)						
Antihypertensives	1642 (18.08%)	43 807 (12.06%)	0.169	736 (15.26%)	619 (12.84%)	0.070
Antilipemic agents	3534 (38.92%)	150 081 (41.33%)	0.049	1687 (34.99%)	1382 (28.66%)	0.136
Long-term and current insulin use	2798 (30.81%)	64 968 (17.89%)	0.305	999 (20.72%)	805 (16.69%)	0.103
Ocular covariates, No. (%)						
Exudative AMD	115 (1.27%)	2431 (0.67%)	0.061	47 (0.98%)	42 (0.87%)	0.011
Nonexudative AMD	65 (0.72%)	4550 (1.25%)	0.054	34 (0.71%)	40 (0.83%)	0.014
Age-related cataract	3660 (40.30%)	47 972 (13.21%)	0.643	1287 (26.69%)	1277 (26.48%)	0.005
Other cataract	1842 (20.28%)	26 158 (7.20%)	0.387	670 (13.90%)	664 (13.77%)	0.004
Glaucoma	1467 (16.16%)	31 427 (8.66%)	0.229	654 (13.56%)	648 (13.44%)	0.004
Lattice degeneration	26 (0.29%)	1228 (0.34%)	0.009	10 (0.21%)	10 (0.21%)	< 0.001
Stages of retinopathy, No. %						
T2DM with unspecified DR	4731 (52.10%)	12 693 (3.50%)	1.291	1814 (37.62%)	1983 (41.12%)	0.072
T2DM with mild NPDR	1306 (14.38%)	1245 (0.34%)	0.558	145 (3.01%)	130 (2.70%)	0.019
T2DM with moderate NPDR	882 (9.71%)	222 (0.06%)	0.459	76 (1.58%)	58 (1.20%)	0.032
T2DM with severe NPDR	938 (10.33%)	75 (0.02%)	0.478	55 (1.14%)	34 (0.71%)	0.046
T2DM with PDR	6595 (72.62%)	3094 (0.85%)	2.230	2391 (49.59%)	2446 (50.73%)	0.023

AA = African American; AMD = age-related macular degeneration; BMI = body mass index; COPD = chronic obstructive pulmonary disease; DR = diabetic retinopathy; HbA1c = hemoglobin A1c; No. = number/frequency; NPDR = nonproliferative diabetic retinopathy; PDR = proliferative diabetic retinopathy; SD = standard deviation; Std. Diff. = standardized difference; T2DM = type 2 diabetes mellitus.

### DR Requiring Vitrectomy vs. Diabetes without Retinopathy

**Figure 2:**
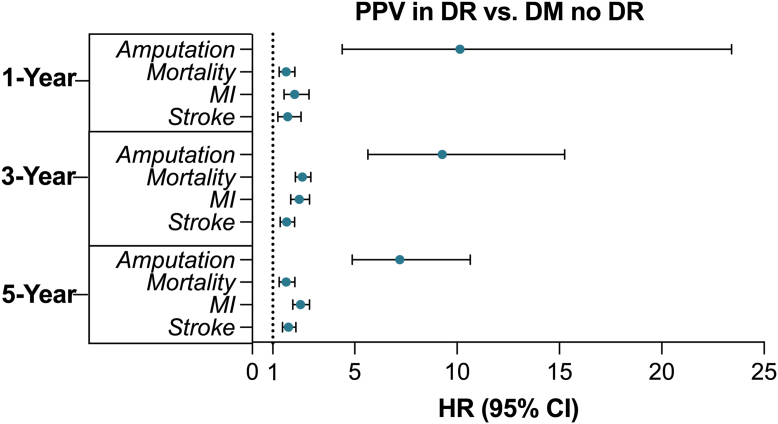
Forest plot of HR at 1, 3, and 5 years for mortality and systemic outcomes between patients with type 2 diabetes with DR undergoing PPV compared with those with diabetes and no DR. CI = confidence interval; DM = diabetes mellitus; DR = diabetic retinopathy; HR = hazard ratio; MI = myocardial infarction; PPV = pars plana vitrectomy.

Table 2 describes the baseline characteristics and matching inputs and results for the comparison between T2DM patients who underwent PPV for DR and those with DM but no DR. The mean (standard deviation) follow-up in days at the end of the 5-year window was 1147.7 (684.3) days in the PPV cohort and 877.0 (645.4) days in the DM with no DR cohort. Patients with DR requiring PPV showed a substantially higher risk of negative systemic health events than diabetics without DR. One year after the index date, HRs for mortality (1.65; 95% CIs: 1.31, 2.08), MI (2.07; 95% CIs: 1.55, 2.77), stroke (1.73; 95% CIs: 1.25, 2.38), and amputation (10.14; 95% CIs: 4.40, 23.41) were all significantly higher in the PPV group. Comparative risk remained significantly elevated for each systemic event in the 3-year analysis, with HR for mortality at 2.45 (95% CIs: 2.10, 2.85), MI at 2.29 (95% CIs: 1.88, 2.80), stroke at 1.68 (95% CIs: 1.37, 2.06), and amputation at 9.28 (95% CIs: 5.65, 15.26). In the 5-year analysis, HRs were significantly elevated for morality, at 2.40 (95% CI: 2.11, 2.73), MI, at 2.35 (95% CI: 1.98, 2.80), stroke, at 1.77 (95% CI: 1.47, 2.13), and amputation, at 7.21 (95% CI: 4.88, 10.65) (Fig 2).

**Table 2:** Propensity Score Matching Results between Patients with Type 2 Diabetes with DR Requiring Vitrectomy and DM without DR

Characteristics	Before Matching		Std. Diff.	After Matching		Std. Diff.
	DR Requiring Vitrectomy (n = 8928)	DM without DR (n = 92 645)		DR Requiring Vitrectomy (n = 8478)	DM without DR (n = 8478)	
Age, mean (SD)	54.5 (12.2)	63.0 (14.4)	0.635	54.9 (12.1)	54.4 (18.2)	0.032
Gender, No. (%)						
Male	4627 (51.83%)	37 132 (40.08%)	0.237	4316 (50.91%)	4276 (50.44%)	0.009
Female	4039 (45.24%)	46 829 (50.55%)	0.106	3900 (46.00%)	3959 (46.70%)	0.014
Unknown	262 (2.94%)	8684 (9.37%)	0.270	262 (3.09%)	243 (2.87%)	0.013
Ethnicity, No. (%)						
Non-Hispanic or Latino	4777 (53.51%)	56 225 (60.69%)	0.146	4607 (54.34%)	4799 (56.61%)	0.046
Hispanic or Latino	2310 (25.87%)	14 794 (15.97%)	0.245	2148 (25.34%)	2050 (24.18%)	0.027
Unknown ethnicity	1841 (20.62%)	21 626 (23.34%)	0.066	1723 (20.32%)	1629 (19.21%)	0.028
Race, No. (%)						
White	4406 (49.35%)	50 508 (54.52%)	0.104	4258 (50.22%)	4419 (52.12%)	0.038
Black or AA	1958 (21.93%)	18 552 (20.03%)	0.047	1864 (21.99%)	1846 (21.77%)	0.005
Asian	271 (3.04%)	4178 (4.51%)	0.077	265 (3.13%)	294 (3.47%)	0.019
American Indian or Alaskan Native	68 (0.76%)	480 (0.52%)	0.031	67 (0.79%)	66 (0.78%)	0.001
Native Hawaiian or Pacific Islander	151 (1.69%)	752 (0.81%)	0.079	139 (1.64%)	119 (1.40%)	0.019
Other race	766 (8.58%)	4969 (5.36%)	0.127	716 (8.45%)	669 (7.89%)	0.020
Unknown race	1308 (14.65%)	13 206 (14.25%)	0.011	1169 (13.79%)	1065 (12.56%)	0.036
Comorbidities, No. (%)						
Essential hypertension	5170 (57.91%)	77 475 (83.63%)	0.590	4996 (58.99%)	4950 (58.38%)	0.011
Hyperlipidemia	2957 (33.12%)	66 559 (71.84%)	0.841	2933 (34.60%)	2929 (34.55%)	0.001
Chronic kidney disease	2376 (26.61%)	20 236 (21.84%)	0.111	2154 (25.41%)	2145 (25.30%)	0.002
COPD	269 (3.01%)	12 136 (13.10%)	0.377	269 (3.17%)	293 (3.46%)	0.016
Alcohol abuse	157 (1.76%)	4231 (4.57%)	0.161	155 (1.83%)	162 (1.91%)	0.006
Nicotine dependence	768 (8.60%)	18 489 (19.96%)	0.329	753 (8.88%)	752 (8.87%)	<0.001
BMI <18.5	145 (1.62%)	2055 (2.22%)	0.043	144 (1.70%)	165 (1.95%)	0.018
BMI ≥18.5, <25	871 (9.76%)	11 100 (11.98%)	0.072	823 (9.71%)	862 (10.17%)	0.015
BMI ≥25, <30	1445 (16.18%)	21 480 (23.19%)	0.177	1375 (16.22%)	1354 (15.97%)	0.007
BMI ≥30	1967 (22.03%)	30 430 (32.85%)	0.244	1880 (22.18%)	1887 (22.26%)	0.002
Laboratory value, No (%)						
HbA1C ≥6.5	3594 (40.26%)	63 382 (68.41%)	0.59	3500 (41.28%)	3583 (42.26%)	0.020
HbA1C <6.5	1155 (12.94%)	56 878 (61.39%)	1.159	1152 (13.59%)	1101 (12.99%)	0.018
Medication, No. (%)						
Antihypertensives	1642 (18.39%)	18 168 (19.61%)	0.031	1523 (17.96%)	1550 (18.28%)	0.008
Antilipemic agents	3534 (39.58%)	68 959 (74.43%)	0.752	3477 (41.01%)	3487 (41.13%)	0.002
Long-term and current insulin use	2700 (30.24%)	26 650 (28.77%)	0.324	2565 (30.26%)	2644 (31.19%)	0.020
Ocular covariates, No. (%)						
Exudative AMD	115 (1.29%)	593 (0.64%)	0.066	94 (1.11%)	94 (1.11%)	<0.001
Nonexudative AMD	65 (0.73%)	2312 (2.50%)	0.140	65 (0.77%)	80 (0.94%)	0.019
Age-related cataract	3585 (40.16%)	29 733 (32.09%)	0.168	3265 (38.51%)	3250 (38.34%)	0.004
Other cataract	1798 (20.14%)	14 773 (15.95%)	0.109	1625 (19.17%)	1648 (19.44%)	0.007
Glaucoma	1440 (16.13%)	13 538 (14.61%)	0.042	1350 (15.92%)	1404 (16.56%)	0.017
Lattice degeneration	26 (0.29%)	913 (0.99%)	0.087	26 (0.31%)	32 (0.38%)	0.012

AA = African American; AMD = age-related macular degeneration; BMI = body mass index; COPD = chronic obstructive pulmonary disease; DM = diabetes mellitus; DR = diabetic retinopathy; HbA1c = hemoglobin A1c; No. = number/frequency; SD = standard deviation; Std. Diff. = standardized difference.

### DR Requiring Vitrectomy vs. Healthy Controls

Baseline cohort characteristics and PSM data for the comparison between patients with T2DM requiring diabetic PPV and healthy patients are shown in Table S3 (available at www.ophthalmologyscience.org). The mean (standard deviation) follow-up in days at the end of the 5-year window was 1146.0 (684.4) days in the PPV cohort and 947.4 (688.5) days in the healthy control cohort. The HR for each of the health events we examined is significantly higher in the PPV group compared with the healthy patients. Analysis at 1 year yielded the following HRs: 1.66 (95% CI: 1.32, 2.08) for mortality, 3.04 (95% CI: 2.18, 4.22) for MI, 2.30 (95% CI: 1.62, 3.26) for stroke, and 20.22 (95% CI: 6.36, 64.34) for amputation. In the 3-year analysis, HRs were significantly elevated for mortality at 2.35 (95% CI: 2.03, 2.72), MI at 3.37 (95% CI: 2.70, 4.21), stroke at 2.75 (95% CI: 2.17, 3.49), and amputation at 32.07 (95% CI: 13.19, 77.99). At 5 years, HRs remained significant for mortality at 2.44 (95% CI: 2.16, 2.76), MI at 3.51 (95% CI: 2.90, 4.26), stroke at 2.61 (95% CI: 2.14, 3.20), and amputation at 43.26 (95% CI: 17.85, 104.83). For all analyzed outcomes, the HR increased from 1 to 5 years.

## Discussion

In a nationwide database study of patients with T2DM undergoing diabetic PPV, we quantified the risk of mortality and adverse systemic events in comparison to those not requiring PPV for DR. We found that these patients have an approximately 30% (HR = 1.28) higher risk of mortality at 5 years compared with those with DR not requiring PPV. Moreover, the risks of MI, stroke, and amputation are elevated by approximately 50%, 54%, and 110%, respectively, at 5 years in the same comparison. Likewise, in comparisons of patients requiring PPV for complications of DR to both patients with DM without DR and healthy patients, 1-, 3-, and 5-year HRs were significantly elevated across all outcomes. These findings indicate a substantial burden of systemic vascular complications in patients with advanced diabetic eye disease requiring surgical intervention, highlighting the need for comprehensive management strategies that address both ocular and systemic health risks in this population.

While our study is not the first to examine mortality after diabetic PPV, we believe it to be definitive. Existing publications have shown decreased survival rates after diabetic PPV, with reported 1 year rates of 96%^28^ and 99%^33^ and 5 year rates in the range of 68% to 86%.^27^, ^28^, ^29^, ^30^, ^31^, ^32^, ^33^, ^34^, ^35^ Our analysis provides advancement upon the existing literature on the grounds of sample size and analytical design. Each prior publication includes between 73 and 552 patients, whereas the 3 comparisons we conducted feature sample sizes of 4822, 8478, and 8817 patients after PSM. Regarding analytical design, prior publications generally analyzed survival rates in a single diabetic PPV cohort without comparing outcomes to patients not undergoing PPV. Only 1 of these studies features direct comparison to another defined cohort.^32^ This aspect of study design, shared in our work, enables cohort matching and the use of comparative ratios to report results. Lux et al^32^ compared the 5 year survival rates of 124 Danish patients who underwent diabetic PPV to “standard diabetes” and “general population” groups, utilizing matching on the bases of age and gender. In this study, the diabetic PPV group had a 79% survival rate at 5 years and the standardized mortality ratios between this cohort and “standard diabetes” and “general population” groups were 1.65 and 2.86, respectively.^32^ The cohorts in this study were not matched for comorbidities associated with diabetes, such as hypertension, hyperlipidemia, and obesity.^39^^,^^40^ Although differences in cohort and experimental design preclude direct comparison of these data to our own, both studies support an association between diabetic PPV and increased risk of mortality. In our study, we conducted PSM on the basis of demographics, comorbidities, medication, and laboratory values, minimizing the effects of confounding variables. Given its comparatively large sample size and extensive matching, our study contributes valuable characterization of the association between undergoing PPV and increased risk of mortality.

In addition, there is a paucity of information regarding the risk of other serious systemic events after diabetic PPV. Although prior research shows an increased risk of MI,^14^^,^^16^, ^17^, ^18^, ^19^, ^20^ stroke,^21^, ^22^, ^23^ and amputation^24^, ^25^, ^26^ in patients with DR, data reporting the risk of these events in the postdiabetic PPV population are limited. In a study by Uchio et al of 73 Japanese patients who underwent PPV for complications of DR, 13 patients were deceased at 5 years. In this subset, 5 patients died from heart failure (possibly related to MI), and 3 patients died from cerebral infarction, signifying the 2 most common causes of death.^30^ We encountered no other published data describing the risk of MI, stroke, or amputation in the postdiabetic PPV population, signaling a gap in the existing literature. We believe these data, as provided by our study, are of high utility for developing a more comprehensive understanding of the health risks of these patients.

### Limitations

We do acknowledge several limitations within our study. Given that our analysis was conducted using a database of prior records, our results are dependent on accurate diagnosis and appropriate management of pathology. Another inherent limitation is sampling bias, as patients with more severe disease are more likely to present for medical care, creating a risk for overrepresentation in data. We attempted to minimize any discrepancy in exposure to care by PSM for ocular conditions not related to diabetes and requiring a record of multiple visits in the DM with no DR and healthy cohorts. Because the data utilized in our analyses were extracted from health care organizations in real-time, there was slight variation in the size of the diabetic PPV cohort between comparisons. The tables describing cohort characteristics and PSM results for each comparison reflect this variance. In addition, the database utilized in our analysis only includes information from health care organizations in the United States, so our data are most applicable to patients in this nation. It was important to utilize only electronic health record data from the United States given the importance of CPT codes in our analysis, which are primarily used in the United States.^36^^,^^37^ Despite extensive PSM, our results remain subject to confounding variables not accounted for in our process. Given that different types of DM are driven by distinct pathophysiology and accompanied by differing health risks, we opted to analyze only patients with DR related to T2DM.^38^ Therefore, our results are only generalizable to this patient population. Finally, an additional limitation applying to all studies of this subject, first noted by Gollamundi et al,^29^ is that some patients with advanced diabetic eye disease may be unable to undergo PPV due to logistical obstacles, such as financial considerations or systemic pathology rendering them ineligible for anesthesia. These individuals, despite having severe ocular disease, would remain in the non-PPV cohort, and their exclusion from the PPV cohort may dilute the apparent differences in risk between cohorts.

While our study identified an association between advanced diabetic eye disease and systemic health risks in patients with T2DM, future projects could build upon our findings by evaluating patients with type 1 DM or other subtypes, for which our dataset included too few cases for reliable analyses. Further, parallel analyses categorizing patients by ocular findings, such as tractional retinal detachment or vitreous hemorrhage, rather than by surgical status alone, may offer additional value for systemic risk stratification. Finally, while the TriNetX database enabled large-scale cohort analysis in our study, future investigations could examine this topic using alternative clinical databases to enhance the reliability, validity, and generalizability of the findings.

In conclusion, this multicenter database study quantified the increased risk of mortality and major systemic health events in patients with T2DM requiring PPV for advanced DR compared with those with DR who did not need PPV. We found that these patients had an approximately 30% higher risk of death, and a 50%, 54%, and 110% higher risk of MI, stroke, and amputation, respectively. Our findings underscore the need for monitoring and management of systemic health in patients with diabetes undergoing PPV for advanced DR. The ophthalmologist’s contribution to this effort may include communicating these risks to patients, encouraging close follow-up with primary care and specialty providers, and corresponding with the rest of the care team to optimize systemic management.
