# Supplementary material for: Risk of Systemic Health Events and Mortality After Vitrectomy for Diabetic Retinopathy in Patients with Type 2 Diabetes
Source: Ophthalmol Sci. 2025 Jul 7;5(6):100880. doi: 10.1016/j.xops.2025.100880 (PMC12363565; doi:10.1016/j.xops.2025.100880)
Supplement: Table S1 [file mmc1.pdf]

Table S1: Coding Utilized for Cohort Assembly

	CPT Code, ICD-10 Code, SNOMED Code, or TriNetX Categorization
Vitrectomy for Complication of Diabetic Retinopathy	67040: Vitrectomy, mechanical, pars plana approach; with endolaser panretinal photocoagulation<br>67041: Vitrectomy, mechanical, pars plana approach; with removal of pre-retinal cellular membrane (eg, macular pucker)<br>67042: Vitrectomy, mechanical, pars plana approach; with removal of internal limiting membrane of retina (eg, for repair of macular hole, diabetic macular edema), includes, if performed, intraocular tamponade (air, gas or silicone oil)<br>67113: Repair of complex retinal detachment (eg, proliferative vitreoretinopathy, stage C-1 or greater, diabetic traction retinal detachment, retinopathy of prematurity, retinal tear of greater than 90 degrees), with vitrectomy and membrane peeling, including, when performed, air, gas, or silicone oil tamponade, cryotherapy, endolaser photocoagulation, drainage of subretinal fluid, scleral buckling, and/or removal of lens
Vitreous Hemorrhage	H43.1: Vitreous hemorrhage
Tractional RD	H33.4: Traction detachment of retina
Rhegmatogenous RD	H33.0: Retinal detachment with retinal break
Diabetic Retinopathy	E11.31: Type 2 diabetes mellitus with unspecified diabetic retinopathy<br>E11.32: Type 2 diabetes mellitus with mild nonproliferative diabetic retinopathy<br>E11.33: Type 2 diabetes mellitus with moderate nonproliferative diabetic retinopathy<br>E11.34: Type 2 diabetes mellitus with severe nonproliferative diabetic retinopathy<br>E11.35: Type 2 diabetes mellitus with proliferative diabetic retinopathy
Diabetes Mellitus	E11: Type 2 diabetes mellitus
Eye Exam	Z01.0: Encounter for examination of eyes and vision<br>Z13.5: Encounter for screening for eye and ear disorders<br>722161008: Diabetic retinal eye exam
Visit	TriNetX Categorization: Visit
Healthy Visit	Z00.00: Encounter for general adult medical examination without abnormal findings

Note: CPT, Common Procedural Terminology; ICD, International Classification of Disease; SNOMED, Systematized Nomenclature of Medicine; RD, Retinal Detachment
