# Supplementary material for: Risk of Systemic Health Events and Mortality After Vitrectomy for Diabetic Retinopathy in Patients with Type 2 Diabetes
Source: Ophthalmol Sci. 2025 Jul 7;5(6):100880. doi: 10.1016/j.xops.2025.100880 (PMC12363565; doi:10.1016/j.xops.2025.100880)
Supplement: Table S2 [file mmc2.pdf]

Table S2. Coding Utilized for Systemic Health Outcomes

	**CPT Code, ICD-10 Code, or TriNetX Categorization**
Mortality	TriNetX Categorization: Deceased<br>R99: Ill-defined and unknown cause of mortality
Myocardial Infarction	I21: Acute myocardial infarction<br>I22: Subsequent ST elevation (STEMI) and non-ST elevation (NSTEMI) myocardial infarction
Stroke	I63: Cerebral infarction
Amputation	26910: Amputation, metacarpal, with finger or thumb (ray amputation), single, with or without interosseous transfer<br>26951: Amputation, finger or thumb, primary or secondary, any joint or phalanx, single, including neurectomies; with direct closure<br>26952: Amputation, finger or thumb, primary or secondary, any joint or phalanx, single, including neurectomies; with local advancement flaps (V-Y, hood)<br>27880: Amputation, leg, through tibia and fibula<br>27881: Amputation, leg, through tibia and fibula; with immediate fitting technique including application of first cast<br>27882: Amputation, leg, through tibia and fibula; open, circular (guillotine)<br>27884: Amputation, leg, through tibia and fibula; secondary closure or scar revision<br>27886: Amputation, leg, through tibia and fibula; re-amputation<br>28800: Amputation, foot; midtarsal (eg, Chopart type procedure)<br>28805: Amputation, foot; transmetatarsal<br>28810: Amputation, metatarsal, with toe, single<br>28820: Amputation, toe; metatarsophalangeal joint<br>28825: Amputation, toe; interphalangeal joint

Note: CPT, Common Procedural Terminology; ICD, International Classification of Disease
