# Supplementary material for: Risk of Systemic Health Events and Mortality After Vitrectomy for Diabetic Retinopathy in Patients with Type 2 Diabetes
Source: Ophthalmol Sci. 2025 Jul 7;5(6):100880. doi: 10.1016/j.xops.2025.100880 (PMC12363565; doi:10.1016/j.xops.2025.100880)
Supplement: Table S3 [file mmc3.pdf]

Table S3. Propensity Score Matching Results Between Type 2 Diabetes Patients with DR requiring Vitrectomy and Healthy Patients.

Characteristics	Before Matching			After Matching		
	DR requiring Vitrectomy (n= 8,928)	Healthy (n= 3,264,709)	Std. Diff.	DR requiring Vitrectomy (n= 8,817)	Healthy (n= 8,817)	Std. Diff.
**Age, mean (SD)**	54.5 (12.2)	50.8 (17.6)	0.240	54.5 (12.2)	58.6 (15.5)	0.294
**Gender, No. (%)**						
Male	4,627 (51.83%)	1,195,067 (36.61%)	0.310	4,544 (51.54%)	4,679 (53.07%)	0.031
Female	4,039 (45.24%)	1,917,835 (58.74%)	0.273	4,011 (45.49%)	3,814 (43.26%)	0.045
Unknown	262 (2.94%)	151,807 (4.65%)	0.090	262 (2.97%)	324 (3.68%)	0.039
**Ethnicity, No. (%)**						
Non-Hispanic/Latino	4,777 (53.51%)	2,359,008 (72.26%)	0.396	4,766 (54.06%)	4,705 (53.36%)	0.014
Hispanic/Latino	2,310 (25.87%)	235,519 (7.21%)	0.519	2,214 (25.11%)	1,870 (21.21%)	0.093
Unknown Ethnicity	1,841 (20.62%)	670,182 (20.53%)	0.002	1,837 (20.84%)	2,242 (25.43%)	0.109
**Race, No. (%)**						
White	4,406 (49.35%)	2,281,856 (69.90%)	0.428	4,369 (49.55%)	4,199 (47.62%)	0.039
Black or AA	1,958 (21.93%)	385,934 (11.82%)	0.272	1,945 (22.06%)	2,057 (23.33%)	0.030
Asian	271 (3.04%)	127,483 (3.91%)	0.048	271 (3.07%)	284 (3.22%)	0.008
American Indian or Alaskan Native	68 (0.76%)	8,030 (0.25%)	0.073	66 (0.75%)	55 (0.62%)	0.015
Native Hawaiian or Pacific Islander	151 (1.69%)	11,224 (0.34%)	0.134	151 (1.71%)	170 (1.93%)	0.016
Other Race	766 (8.58%)	103,578 (3.17%)	0.231	731 (8.29%)	724 (8.21%)	0.003
Unknown Race	1,308 (14.65%)	346,604 (10.62%)	0.122	1,284 (14.56%)	1,328 (15.06%)	0.014
**Comorbidities, No. (%)**						
Essential Hypertension	5,170 (57.91%)	1,058,532 (32.42%)	0.530	5,060 (57.39%)	5,215 (59.15%)	0.036
Hyperlipidemia	2,957 (33.12%)	917,432 (28.10%)	0.109	2,909 (32.99%)	2,966 (33.64%)	0.014
Chronic Kidney Disease	2,376 (26.61%)	124,324 (3.81%)	0.670	2,267 (25.71%)	2,325 (26.37%)	0.015
COPD	269 (3.01%)	121,699 (3.73%)	0.040	269 (3.05%)	311 (3.53%)	0.027
Alcohol Abuse	157 (1.76%)	83,246 (2.55%)	0.055	154 (1.75%)	139 (1.58%)	0.013
Nicotine Dependence	768 (8.60%)	356,509 (10.92%)	0.078	762 (8.64%)	798 (9.05%)	0.014
BMI ≥25	2,790 (31.25%)	1,333,763 (40.85%)	0.201	2,764 (31.35%)	2,641 (29.95%)	0.030
BMI <25	928 (10.39%)	855,226 (26.20%)	0.418	917 (10.40%)	862 (9.77%)	0.021

**Medication, No. (%)**						
Antihypertensives	1,642 (18.40%)	219,117 (6.71%)	0.358	1,569 (17.80%)	1,621 (18.39%)	0.015
Antilipemic Agents	3,534 (39.58%)	694,715 (21.28%)	0.406	3,439 (39.0%)	3,387 (38.41%)	0.012
**Ocular Covariates, No. (%)**						
Exudative AMD	115 (1.29%)	5,858 (0.17%)	0.130	105 (1.19%)	110 (1.25%)	0.005
Non-exudative AMD	65 (0.73%)	14,976 (0.46%)	0.035	65 (0.74%)	77 (0.87%)	0.015
Age-related Cataract	3,585 (40.16%)	154,992 (4.75%)	0.937	3,474 (39.40%)	3,758 (42.62%)	0.066
Other Cataract	1,798 (20.14%)	95,503 (2.93%)	0.560	1,720 (19.51%)	1,860 (21.10%)	0.040
Glaucoma	1,440 (16.13%)	95,996 (2.94%)	0.461	1,393 (15.80%)	1,549 (17.57%)	0.048

Note: DR, Diabetic Retinopathy; Std. Diff., standardized difference; SD, standard deviation; No., number/frequency; AA, African American; COPD, Chronic Obstructive Pulmonary Disease; BMI, Body Mass Index; AMD, Age-related Macular Degeneration
